# Synthesis and characterization of VO_2_-based thermochromic thin films for energy-efficient windows

**DOI:** 10.1186/1556-276X-6-301

**Published:** 2011-04-07

**Authors:** Carlos Batista, Ricardo M Ribeiro, Vasco Teixeira

**Affiliations:** 1 Department of Physics, University of Minho, Campus de Gualtar, 4710-057 Braga, Portugal

## Abstract

Thermochromic VO_2 _thin films have successfully been grown on SiO_2_-coated float glass by reactive DC and pulsed-DC magnetron sputtering. The influence of substitutional doping of V by higher valence cations, such as W, Mo, and Nb, and respective contents on the crystal structure of VO_2 _is evaluated. Moreover, the effectiveness of each dopant element on the reduction of the intrinsic transition temperature and infrared modulation efficiency of VO_2 _is discussed. In summary, all the dopant elements--regardless of the concentration, within the studied range-- formed a solid solution with VO_2_, which was the only compound observed by X-ray diffractometry. Nb showed a clear detrimental effect on the crystal structure of VO_2_. The undoped films presented a marked thermochromic behavior, specially the one prepared by pulsed-DC sputtering. The dopants effectively decreased the transition of VO_2 _to the proximity of room temperature. However, the IR modulation efficiency is markedly affected as a consequence of the increased metallic character of the semiconducting phase. Tungsten proved to be the most effective element on the reduction of the semiconducting-metal transition temperature, while Mo and Nb showed similar results with the latter being detrimental to the thermochromism.

## Introduction

Solar control coatings are a technology of growing interest due to the necessity of improving the energy efficiency of buildings, with a view to avoiding excessive energy consumption due to cooling systems during summer. The latest approach is based on the use of thermochromic coatings on the so-called smart windows. These coatings possess the ability of actively changing their optical properties as a consequence of a reversible structural transformation when going through a critical temperature.

Vanadium dioxide is an example of a thermochromic material which is a promising candidate for this kind of application as proposed by Granqvist [1]. The change on its optical and also electrical properties takes place at approximately 68°C as a result of a first-order structural transition, going from a monoclinic to a tetragonal phase upon heating [2,3]. The atomic displacements driven by the structural transition are accompanied by a redistribution of the electronic charge in the crystal lattice, which in turn changes the nature of the interatomic bonding [4]. The low-temperature semiconducting phase which is transparent to radiation in the visible and infrared spectral ranges maximizes the heating because of blackbody radiation, while the metallic high-temperature phase filters the infrared radiation and maintains at the same time the transparency required, in the visible range, to maintain an environment of natural light. In order to achieve a reasonable transparency (transmittance, 40-60%) in the visible range and at the same time an acceptable IR modulation efficiency, the VO_2 _films must not exceed thicknesses in the order of 100-150 nm [5], and combined with anti-reflection coatings, the transparency can be further improved [6,7]. To obtain window coatings with controlled thicknesses in the nanometer range, atomistic processes such as magnetron sputtering are well suited to fulfill the condition. A semiconductor-metal transition temperature of 68°C is too high for this application and must therefore be reduced. At present, there are two approaches to reduce the transition temperature, the substitution of part of the vanadium cations by other metals such as tungsten [8-14], molybdenum [15-18], or niobium [16,19,20], or the substitution of part of the oxygen anions by other elements, e.g., fluorine [21].

In this study, we compare magnetron-sputtered VO_2 _thin films prepared with different doping elements such as W, Mo, and Nb and different doping concentrations. We report on the influence of each element and respective concentrations on the crystal structure of the films, optical/thermochromic performance and effectiveness on the reduction of the semiconductor-metal transition from 68°C to room temperature, envisaging the application on energy-efficient windows.

## Experimental details

The vanadium dioxide films were reactively deposited onto SiO_2_-coated float glass substrates by DC and pulsed-DC magnetron sputtering from a high purity (99.95%) metallic vanadium target in a given oxygen/argon atmosphere. Before the deposition, the vacuum chamber was evacuated down to a pressure of about 3 × 10^-5 ^mbar. A pre-sputtering of the metal target was carried out before each deposition during 10 min, in the same conditions as for film preparation, but in an oxygen-free atmosphere. This procedure ensures an oxide-free metallic surface for each deposition. For the deposition of the films, both oxygen and argon were introduced into the chamber separately through two gas mass flow controllers. The deposition parameters chosen to deposit the three sets of films are summarized in Table 1. The doping of the films was done by placing a number of high-purity dopant metal pieces in a concentric positioning over the round vanadium target so that both elements could be co-sputtered allowing a homogeneous dispersion of the dopant elements in the film. In order to obtain films with different dopant concentrations, the number of dopant pieces has been either varied or moved along the target surface.

**Table 1:** Processing conditions used for depositing the VO_2 _films

	W- and Mo-doped films	Nb-doped films
Base pressure (mbar)	3 × 10^-5^	3 × 10^-5^
Work pressure (mbar)	4 × 10^-3^	1 × 10^-3^
Oxygen/argon ratio (%)	14.3	50
Total gas flow (sccm)	19.2	6
DC current (A)	0.5	-
Pulsed-DC current (A)	-	0.58
Frequency (kHz)	-	10
Reverse time (μs)	-	5
Substrate temperature (°C)	450	450
Deposition time (min)	5	3

The actual doping concentration in the films has been determined by X-ray photoelectron spectroscopy which permitted to assess the elemental composition of the films. The structural characterization has been done by X-ray diffractometry (XRD) using a X-ray diffractometer operating with a continuous scan of Cu K_α1 _radiation with λ = 1.54056 Å. The optical/thermochromic behavior has been evaluated in an optical spectrophotometer (Shimadzu UV-3101PC) with an embedded sample heating-cooling cell. It has been done by measuring the spectral normal transmittance at the UV-Vis-near-infrared (NIR) range, from 250 to 2500 nm, under and above the transition temperature. The determination of the transition temperature was carried out by evaluating the optical transmittance change with temperature at a given NIR wavelength, in this case at λ = 2500 nm. The transition temperatures were then estimated by determining the first derivative of both curves of the hysteresis loops (heating and cooling) and considering the mean value.

## Results and discussion

### Structural characterization

**Figure 1:**
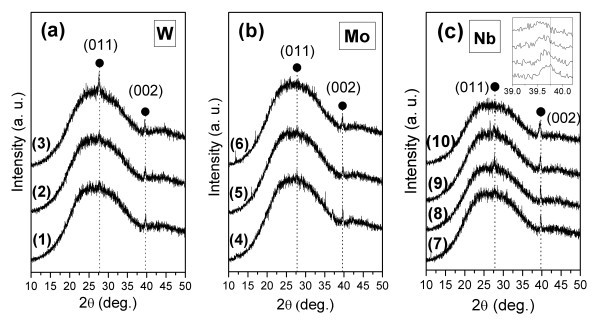
**XRD spectra of VO_2 _films deposited by (a1-a3, b4-b6) DC and (c7-c10) pulsed-DC sputtering, doped with different dopant element and contents**: (a1) pure VO_2_, (a2) V_0.97_W_0.03_O_2_, and (a3) V_0.95_W_0.05_O_2_; (b4) V_0.97_Mo_0.03_O_2_, (b5) V_0.94_Mo_0.06_O_2_, and (b6) V_0.89_Mo_0.11_O_2_; (c7) pure VO_2_, (c8) V_0.96_Nb_0.04_O_2_, (c9) V_0.93_Nb_0.07_O_2_, and (c10) V_0.89_Nb_0.11_O_2_.

The crystal structure of the three sets of films has been assessed by XRD, and the obtained diffraction spectra are shown in Figure 1. The XRD patterns show the range where the most significant reflection peaks of VO_2 _appear. The poor signal intensities of the crystallite-reflected plane directions are due to the nanocrystallinity and small thicknesses of the films which are estimated to be around 125 nm, for the chosen processing conditions [5]. Despite the broad shoulder found within 15-40° which is due to the contribution of the amorphous volume of glass substrate, all patterns can be indexed to single-phase VO_2_(M) which holds a monoclinic structure [22]. No reflections were observed attributable to other vanadium oxides or to compounds deriving from the dopant elements, which suggests that a solid solution of vanadium dioxide with dopant homogeneously dispersed is formed. It can be seen in Figure 1a that for the given processing parameters, pure vanadium dioxide reveals a structure preferably oriented in the (002) plane direction, as observed by the peak at 2θ = 39.6°, although some traces of (011) reflection are detectable at 2θ = 27.8°. With addition of tungsten to a certain extent, as seen in pattern (2) for film V_0.97_W_0.03_O_2_, the same preferential crystal orientation is maintained. The film with the highest W content, V_0.95_W_0.05_O_2_, reveals an evident polycrystalline structure in which the (011) plane direction becomes the dominating crystal orientation. This indicates the existence of a critical level of W contents in the VO_2 _solid solution above which the structure becomes more stably oriented along the (011) direction. All the Mo-doped films reveal preferential crystal orientation along the (002) direction for all films regardless of the Mo-doping level, although some traces of crystallites oriented along the (011) and (21-1) directions are barely noticeable at 2θ = 27.8° and 37.0°, respectively. In summary, no significant differences on the crystal structure can be observed in the films with different Mo contents. This is in agreement with results reported for Mo-doped VO_2 _on single crystal sapphire substrates prepared by pulsed laser deposition [23] and RF-sputtered Mo-doped VO_2 _[17] although the latter presents strong (011) preferred orientation. With regard to the VO_2 _films prepared by pulsed-DC sputtering, shown in Figure 1c, the main crystal orientation is again along the (002) direction although the (011) is also noticeable in some of the films. Comparing the patterns among the different Nb contents in the region of the (002) diffraction peak, as seen in the inset, a shifting of the peak to lower angles accompanied by a broadening is observed as the Nb at.% in the film is increased. X-ray diffraction peaks broaden either when crystallites become smaller or if lattice defects such as microstresses, stress gradients, and/or chemical heterogeneities are present in large enough abundance [24]. Peak shift is related to different types of internal stresses and planar faults in the crystal lattice, especially stacking faults or twin boundaries. In this particular case, the peak shifts toward lower diffraction angles, implying an increase of interplanar spacing after Nb doping. These changes on the (002) diffraction peak parameters have not been observed in our previous studies for tungsten [14], molybdenum [18,25], and Indium [25] as dopants in VO_2_.

### Optical analyses

**Figure 2:**
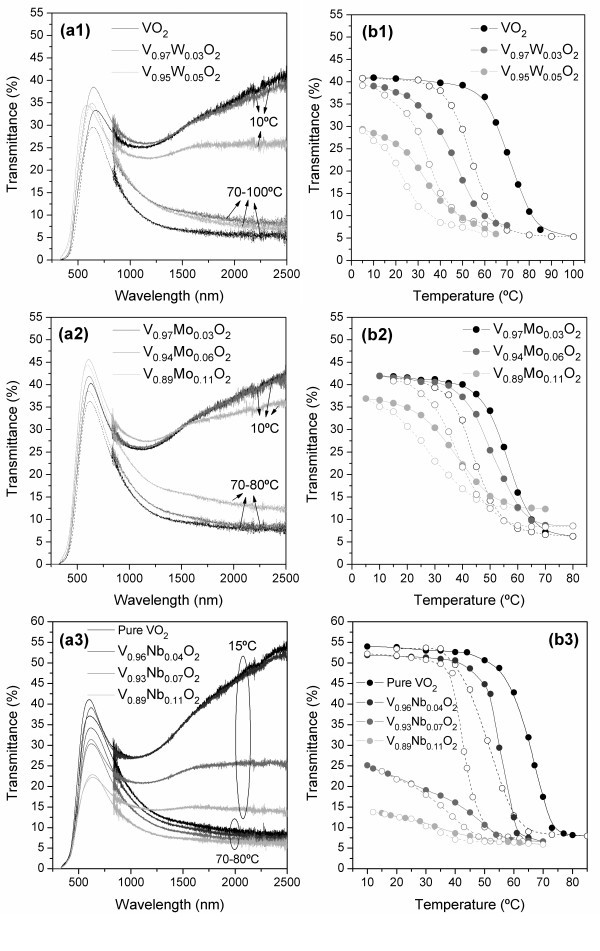
**Optical transmittance spectra of VO_2 _films: (a1-a3) optical transmittance as a function of wavelength, in semiconducting and metallic states; (b1-b3) optical transmittance as a function of temperature obtained at λ = 2500 nm**.

The optical properties of the films have been studied by optical spectrophotometry in the UV-Vis-NIR range, and the obtained results are shown in Figure 2. On the left is shown the optical transmittance as a function of wavelength, and on the right is shown the optical transmittance at λ = 2500 nm as a function of temperature. It can be seen in Figure 2a1 that maximum luminous transmittances of about 30-40% are associated with a sharp thermochromic switch behavior at the NIR spectral range that is reduced by increasing W doping concentrations. The differences regarding the maximum luminous transmittances are mainly due to slight variations in thickness from film to film and not due to a significant influence of tungsten, which is in accordance with that observed by Burkhardt et al. [8]. With increasing W doping concentration up to 5%, the IR modulation efficiency (*T*_s_-*T*_m_) reduced from 35%, for the undoped film down to 23%. Moreover, a slight loss can be observed in the luminous transparency when switching from a semiconducting to a metallic state, which is common in all the films regardless of the dopant element and concentration. The Mo-doped films showed maximum optical transmittances in the visible range from 35 to 45% and decreased IR modulation efficiency from 36 to 25% with increasing substitutional Mo content from 3 to 11%. The infrared modulation efficiency of the pure VO_2 _film prepared by pulsed-DC sputtering, shown in Figure 2a3 was found to be higher than that of VO_2 _prepared by conventional DC sputtering, as seen in Figure 2a1. The use of an asymmetric-bipolar, pulsed DC power supply allows higher sputtering yields by periodically reversing the electrode voltage, thereby neutralizing charge build-up on the target surface during poisoning in the reactive process. In addition, it also reduced the working gas pressure and increased the ion current density. All these factors contribute to a higher ion bombardment during film growth which contributes to an improved film density/crystallinity and enhancement of its properties. The IR modulation efficiency is again affected by the Nb contents in the film, and a marked drop is obvious for Nb over 4 at.%. Above this Nb content, the material starts revealing a very pronounced metal-like character, as demonstrated by the decrease of transparency to IR light of the low-temperature phase. Moreover, the maximum luminous transmittance is around 40%, for pure VO_2_, and progressively decreases down to 22% with the increase of substitutional Nb up to 11 at.% in the VO_2 _solid solution. The decrease in the IR modulation efficiency resulting from doping is mainly due to decrease in the transmittance in the semiconducting state. This decrease is explained by the enhancement of the carrier concentration due to the presence of dopant ion donors [21,26] which also lowers the resistivity of the films [26]. The doping of VO_2 _increased the electron density in the film, which caused the Fermi energy level shift toward the conduction band. Since intrinsic VO_2 _thin film is of *n*-type, introduction of ion donors cause an inevitable degradation of the transmittance (and resistivity) of the semiconducting low-temperature phase. Likewise, it is expected that the enhancement of the carrier concentration would also lower the transmittance at the infrared in the metallic state, which indeed does so in the case of the Nb-doped films, as seen in Figure 2a3. However, W- and Mo-doped films do not show the same trend. Although we were not able to effectively determine crystallite sizes because of poor peak statistics of XRD patterns for the different doped films, it has been shown that doping reduces the crystallite size [27,28]. Therefore, the number of crystallites as well as boundaries volume will increase and contribute to trap charge carriers which will result in loss of the metallic behavior. We speculate that in case of W- and Mo-doped films, this effect could be more marked than that of increase in carrier concentration due to W and Mo donors. Substitution of V^4+ ^by higher valence cations, such as Nb^5+^, W^6+^, and Mo^6+^, give rise to the same V_1-*x*_M*_x_*O_2 _system [2]. According to studies conducted by Tang et al. [29], each added W ion breaks up a V^4+^-V^4+ ^homopolar bond and causes the transfer of two 3*d *electrons to the nearest V ions for charge compensation, forming two new bonds, V^3+^-W^6+ ^and V^3+^-V^4+^. The loss of homopolar V^4+^-V^4+ ^bonding destabilizes the semiconducting phase and lowers the metal-semiconductor transition temperature. As regards W doping, Mo acts in the same way on the reduction of phase transition temperature, i.e., introducing extra electrons in the *d *bands of vanadium which induce a charge transfer from Mo to V [2]. In the case Nb, according to Magariño et al. [20], the Nb^4+ ^ion substitutes the V^4+ ^ion in the V^4+^-V^4+ ^bonding and due to charge transfer a V^3+^-Nb^5+ ^bond is formed.

As observed in Figure 2b1,b2,b3, the semiconductor-metal phase transition exhibits a characteristic thermal hysteresis which is due to latent heat evolved and absorbed during the first-order structural transition [17]. The shifting of the hysteresis loops to lower temperatures as a consequence of the increasing contents of substitutional W in the VO_2 _solid solution is very clearly seen. The resulting transition temperatures determined from the optical transmittance hysteresis loops were adjusted from 63 to 28°C. The addition of Mo or Nb to VO_2 _also affects the hysteresis loops which are also shifted to lower temperatures as the doping concentration increases. Transition temperatures as low as 32 and 34°C were achieved for Mo-doped and Nb-doped films, respectively. The transition temperature (*T*_t_) obtained for the pure VO_2 _film prepared by pulsed-DC sputtering was 59°C, which is lower than that obtained for VO_2 _prepared by DC sputtering, i.e., 63°C. It is known that the transition temperature of pure VO_2 _in thin film form may present reduced values depending on properties, such as stresses, thickness, stoichiometry, structure, grain size, etc. [9,15], which are directly associated to the chosen processing conditions. Pure VO_2 _shows a clear transition region with well-defined semiconducting and metal domains. The doped V_0.96_Nb_0.04_O_2 _film shows a similar hysteresis loop shape but with a clear shift to lower temperatures without any significant loss in the transmission in the semiconducting state. For higher Nb concentrations, there is an obvious degradation of the hysteresis which causes the ambiguous boundaries of the transition The estimated transition temperatures in these cases are not in fact a result of a real reduction in the temperature, which would be given by a shift of the hysteresis, but rather in a reduction of the slope of the transition. In all cases a reduction of the hysteresis width is also observable, which is assumed to result from the reduction in the size of the crystallite distribution with doping [17,21].

**Figure 3:**
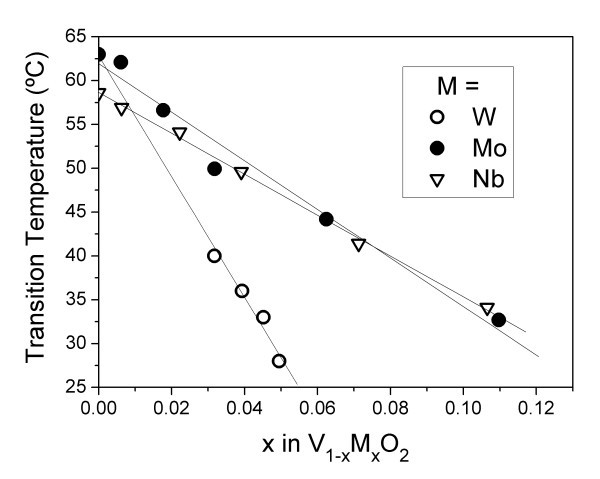
**Relationship between the dopant contents in the film and the resultant semiconductor-metal phase transition temperature**.

The effectiveness of each dopant on the reduction of the semiconducting-metal transition temperature in VO_2 _is compared in Figure 3. All the three elements showed a linear decrease of the transition temperature with the increase in the concentration of substitutional doping element. Tungsten is clearly the most effective dopant element showing a decrease of about 7°C per at.%. Mo and Nb showed nearly the same results, about 3 and 2°C, per at % Mo and Nb, respectively.

## Conclusions

Thermochromic VO_2 _thin films were successfully synthesized by DC and pulsed-DC reactive magnetron sputtering. Different dopant elements, such as tungsten, molybdenum, and niobium, with different doping concentrations were introduced in the VO_2 _solid solution during the film growing by co-sputtering the respective metal dopants, and Vanadium in a reactive O_2_/Ar atmosphere. XRD results showed single phase VO_2_(M) for all the films regardless of dopant element and concentration. The dopants effectively decreased the transition temperature of VO_2 _whereas the thermochromism of the films was markedly affected, especially that in the Nb-doped ones. Nb causes significant amount of defects in the crystal lattice which clearly degrade the optical properties while reducing the semiconductor-metal transition to room temperature.

## Abbreviations

XRD: x-ray diffractometry.

## Competing interests

The authors declare that they have no competing interests.

## Authors' contributions

CB designed the study, carried out the experimental work and draft the manuscript. RR and VT coordinated the study. All authors read and approved the final manuscript.

## References

[B1] Granqvist CG Spectrally Selective Coatings for Energy Efficiency and Solar Applications Phys Scr 1985 32 401 10.1088/0031-8949/32/4/026

[B2] Goodenough JB The two components of the crystallographic transition in VO2 J Solid State Chem 1971 3 490 10.1016/0022-4596(71)90091-0

[B3] Zylbersztejn A Mott NF Metal-insulator transition in vanadium dioxide Phys Rev B 1975 11 4383 10.1103/PhysRevB.11.4383

[B4] Goodenough JB Metallic oxides Prog Solid State Chem 1971 5 145 10.1016/0079-6786(71)90018-5

[B5] Batista C Teixeira V Carneiro J Structural and Morphological Characterization of Magnetron Sputtered Nanocrystalline Vanadium Oxide Films for Thermochromic Smart Surfaces J Nano Res 2008 2 21 10.4028/www.scientific.net/JNanoR.2.21

[B6] Mlyuka NR Niklasson GA Granqvist CG Thermochromic multilayer films of VO2 and TiO2 with enhanced transmittance Solar Energy Mater Solar Cells 2009 93 1685 10.1016/j.solmat.2009.03.021

[B7] Jin P Xu G Tazawa M Yoshimura K Design, formation and characterization of a novel multifunctional window with VO2 and TiO2 coatings Appl Phys A Mater Sci Process 2003 77 455 10.1007/s00339-002-1460-2

[B8] Burkhardt W Christmann T Meyer BK Niessner W Schalch D Scharmann A W- and F-doped VO2 films studied by photoelectron spectrometry Thin Solid Films 1999 345 229 10.1016/S0040-6090(98)01406-0

[B9] Sobhan MA Kivaisi RT Stjerna B Granqvist CG Thermochromism of sputter deposited WxV1-xO2 films Solar Energy Mater Solar Cells 1996 44 451 10.1016/S0927-0248(95)00051-8

[B10] Manning TD Parkin IP Pemble ME Sheel D Vernardou D Intelligent window coatings: Atmospheric pressure chemical vapor deposition of tungsten-doped vanadium dioxide Chem Mater 2004 16 744 10.1021/cm034905y

[B11] Jin P Nakao S Tanemura S Structural and optical characterization of VOx films doped with W by ion implantation Ion Implantation Technology Proceedings, 1998 International Conference 1998 1051

[B12] Parkin IP Manning TD Intelligent thermochromic windows J Chem Educ 2006 83 393 10.1021/ed083p393

[B13] Binions R Piccirillo C Parkin IP Tungsten doped vanadium dioxide thin films prepared by atmospheric pressure chemical vapour deposition from vanadyl acetylacetonate and tungsten hexachloride Surf Coat Technol 2007 201 9369 10.1016/j.surfcoat.2007.03.026

[B14] Batista C Ribeiro R Carneiro J Teixeira V DC sputtered W-doped VO2 thermochromic thin films for smart windows with active solar control J Nanosci Nanotechnol 2009 9 4220 10.1166/jnn.2009.M36 19916434

[B15] Hanlon TJ Coath JA Richardson MA Molybdenum-doped vanadium dioxide coatings on glass produced by the aqueous sol-gel method Thin Solid Films 2003 436 269 10.1016/S0040-6090(03)00602-3

[B16] Manning TD Parkin IP Blackman C Qureshi U APCVD of thermochromic vanadium dioxide thin films - solid solutions V2-xMxO2 (M = Mo, Nb) or composites VO2: SnO2 J Mater Chem 2005 15 4560 10.1039/b510552h

[B17] Jin P Tanemura S V1-xMoxO2 thermochromic films deposited by reactive magnetron sputtering Thin Solid Films 1996 281-282 239 10.1016/0040-6090(96)08641-5

[B18] Batista C Teixeira V Ribeiro RM Synthesis and Characterization of V1-xMoxO2 Thermochromic Coatings with Reduced Transition Temperatures J Nanosci Nanotechnol 2010 10 1393 10.1166/jnn.2010.1873 20352805

[B19] Batista C Carneiro J Ribeiro R Teixeira V Reactive Pulsed-DC Sputtered Nb-doped VO2 coatings for smart thermochromic windows with active solar control J Nanosci Nanotechnol in press 10.1166/jnn.2011.3486 22400299

[B20] Magariño J Tuchendler J D'Haenens JP High-frequency EPR experiments in niobium-doped vanadium dioxide Phys Rev B 1976 14 865

[B21] Burkhardt W Christmann T Franke S Kriegseis W Meister D Meyer BK Niessner W Schalch D Scharmann A Tungsten and fluorine co-doping of VO2 films Thin Solid Films 2002 402 226 10.1016/S0040-6090(01)01603-0

[B22] The International Centre for Diffraction Data (ICDD) Powder Diffraction File 44 252

[B23] Wu ZP Miyashita A Yamamoto S Abe H Nashiyama I Narumi K Naramoto H Molybdenum substitutional doping and its effects on phase transition properties in single crystalline vanadium dioxide thin film J Appl Phys 1999 86 5311 10.1063/1.371519

[B24] Ungár T Microstructural parameters from X-ray diffraction peak broadening Scr Mater 2004 51 777

[B25] Batista C Teixeira V Ribeiro RM Mo and In-doped VO_2 _thermochromic coatings grown by reactive DC magnetron sputtering52nd Annual SVC Technical Conference, Santa Clara, California, USA 2009 451

[B26] Soltani M Chaker M Haddad E Kruzelecky RV Margot J Effects of Ti-W codoping on the optical and electrical switching of vanadium dioxide thin films grown by a reactive pulsed laser deposition Appl Phys Lett 2004 85 1958 10.1063/1.1788883

[B27] Bayard MLF Reynolds TG Vlasse M McKinzie HL RJ Arnott Wold A Preparation and properties of oxyfluoride systems V2O5-xFx and VO2-xFx J Solid State Chem 1971 3 484 10.1016/0022-4596(71)90090-9

[B28] Case FC Influence of ion beam parameters on the electrical and optical properties of ion-assisted reactively evaporated vanadium dioxide thin films J Vac Sci Technol A 1987 5 1762 10.1116/1.574534

[B29] Tang C Georgopoulos P Fine ME Cohen JB Nygren M Knapp GS Aldred A Local atomic and electronic arrangements in WxV1-xO2 Phys Rev B 1985 31 1000 10.1103/PhysRevB.31.1000 9935847

